# Multiplex SERS Detection of Polycyclic Aromatic Hydrocarbon (PAH) Pollutants in Water Samples Using Gold Nanostars and Machine Learning Analysis

**DOI:** 10.1039/d3an00636k

**Published:** 2023-10-05

**Authors:** Supriya Atta, Joy Li, Tuan Vo-Dinh

**Affiliations:** a Fitzpatrick Institute for Photonics, Duke University, Durham, NC 27708, USA; b Department of Biomedical Engineering, Duke University, Durham, NC 27708, USA; c Department of Chemistry, Duke University, Durham, NC 27708, USA

**Keywords:** Gold nanostars, sharp spikes, SERS, machine learning, PAH

## Abstract

Polycyclic aromatic hydrocarbons (PAH) have gained a lot of environmental concern because of their carcinogenic, and mutagenic properties, and they can easily contaminate natural resources such as drinking water and river water. This study presents a simple and sensitive point-of-care SERS detection of PAH combined with machine learning algorithms to predict the PAH content more precisely and accurately in real-life samples such as drinking water and river water. We first synthesized multibranched sharp-spiked surfactant-free gold nanostars (GNS), which can generate strong surface-enhanced Raman scattering (SERS) signal, which was further coated with cetyltrimethylammonium bromide (CTAB) for long-term stability of GNS as well as to trap PAH. We utilized the CTAB-capped GNS for solution-based ‘mix and detect’ SERS sensing of various PAH including pyrene (PY), nitro-pyrene (NP), anthracene (ANT), benzo[a]pyrene (BAP), and triphenylene (TP) spiked in drinking water and river water using a portable Raman module. Very low limits of detection (LOD) were achieved in the nanomolar range for the PAH investigated. More importantly, the detected SERS signal was reproducible for over 90 days of synthesis. Furthermore, we have analyzed the SERS data using artificial intelligence (AI) with machine learning algorithms based the convolutional neural network (CNN) model in order to discriminate the PAH in samples more precisely and accurately. Using a CNN classification model, we have achieved a high prediction accuracy of 90% in the nanomolar detection range and f1 score (harmonic mean of precision and recall) of 94%, using a CNN regression model, achieved a RMSE_conc_=1.07×10^−1^ μM. Overall, our SERS platform can be effectively and efficiently used for the accurate detection of PAH in real-life samples, thus opening up a new, sensitive, selective, and practical approach for point-of-need SERS diagnostic of small molecules in complex practical environments.

## Graphical Abstract

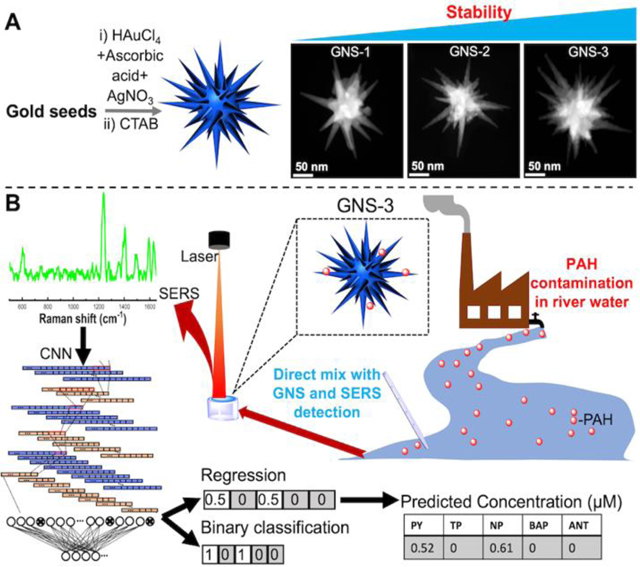

## Introduction

PAH are well-known environmental pollutants due to their mutagenic and carcinogenic properties^1–3^. PAH are generally generated from incomplete combustion of materials such as oil, coal, petrol, or wood^4, 5^. Although PAH are highly hydrophobic, they can dissolve in mg/L concentration range in natural water sources and contaminate water^6, 7^. Therefore, it is important to develop a rapid and on-site detection of PAH to prevent contamination of natural resources. Most conventional analysis techniques for PAH detection are high performance liquid chromatography (HPLC),^8, 9^ and gas chromatography-mass spectrometry (GC-MS)^10–12^. However, these methods are time-consuming and labor-intensive, require heavy instrumentation, and hence they lack practicality for widespread use. Molecular Spectroscopy can provide analytical tools for easy, rapid, sensitive, and in-field detection of PAH. Synchronous fluorescence technique has previously been developed to detect PAH in complex mixtures^13^. More recently, surface-enhanced Raman scattering (SERS) spectroscopy has attracted attention as an analytical technique for rapid, sensitive, label-free, and nondestructive detection of a wide range of analytes.^14–16^ Over three decades our laboratory has been developing various SERS-based nanosystems for many different applications spanning chemical and biological sensing, remote monitoring, work of art analysis, medical diagnostics and therapy (theranostic).^17–22^ Sensitive SERS platforms generally require metallic nanostructures containing multiple number of sharp tips or tiny holes, intermetallic junctions or gaps between metal nanoparticles, which is commonly known as ‘hot spots’, to enhance the electromagnetic field and produce a SERS enhancement factor up to 10^9^-10^10^ orders of magnitude.^14^ In the past decade, various approaches have been developed to use SERS for detection of PAH.^23–32^ For example, Montes-García et al. developed a thin film based on citrate-stabilized Au nanoparticles (NPs) conjugated with ammonium pillar [5]arene (AP[5]A) which can trap PAH into the hydrophobic pocket of AP[5]A^30^. Moreover, Gu et al. introduced 1,10-decanedithiol to capped on silver nanocavity to capture pyrene in silver cavity substrate^33^. However, despite great advances for sensitive PAH detection, most of the reported SERS platforms involve a complex process to produce them and they are not stable for long-term. In contrast, solution-based SERS platform can meet the requirements for point-of-need SERS detection under field conditions. Unfortunately, currently SERS detection systems suffer from very poor sensitivity, which is mainly due to weak affinity of the analytes preventing them from getting too close to the metal surface in order to experience a strong plasmonic effect. We believe that a suitable nanostructure with multiple ‘hot-spots’ can enhance the sensitivity of solution-based platform.

Among different morphologies of nanoparticles, gold nanostars (GNS), which has a spherical core with sharp protruding tips, have attracted strong interest in research community because of their unique shape-dependent optical properties known as localized surface plasmon resonance (LSPR) and can produce highly localized electromagnetic field to enhance the Raman signal of analyte molecules^34–36^. To date, tremendous efforts have been devoted to modifying the morphology of GNS for different applications such as sensing, imaging, and photocatalysis^37–46^. For example, our laboratory first introduced GNS as a SERS probe for detection of small organic molecules^47^. Although there has been great progress to employ GNS for sensing applications, it is still quite challenging to achieve reproducible and sensitive SERS using GNS as GNS has high energetic facets at the tips, which destabilizes the morphology of GNS^48^. Therefore, it is becoming more difficult to achieve consistent and accurate SERS response in complex real-life samples.

Another important parameter for point-of-need SERS detection is to interpret the Raman data more precisely and accurately. The interpretation of Raman spectra is not straightforward because of overlapping peaks of analytes, weak Raman signal, and high background noise. Moreover, it becomes a serious challenge in the presence of external molecules present in complex, real-life samples which can overlap with the characteristic peaks of the target analytes. Multiplexed SERS spectra were conventionally analyzed by identification of unique, characteristic peaks, spectral decomposition ^49^. Identifying characteristic peaks cannot detect analytes with overlapping peaks; spectral decomposition is sensitive to noise and changes in background. In contrast, introducing machine learning algorithms for Raman data preprocessing can extract the Raman signal information more accurately in a complex environment. Various machine learning models including support vector machine ^50^, random forest ^51^, and convolutional neural networks (CNN) ^52^ have been increasingly applied to analyze multiplexed SERS spectra. Compared to other machine learning methods, CNN is more effective in classification ^53^ and regression analysis ^54^ of SERS spectra. CNN has been used to analyze SERS spectra for identification of pathogenic bacteria ^55^, identification and quantification of multiplexed pesticides ^56^. We previously reported CNN as the most effective model for SERS analysis of biomarkers for medical diagnostics ^54^. In this study, we demonstrated the first application of CNN on SERS spectra for classification and quantification of PAH.

We proposed a CTAB capped multi-branched sharp spiked GNS as a SERS platform for trace detection of PAH, which combines the ultra-high SERS properties of GNS and the advantage of CTAB to trap PAH molecules. We have further applied our SERS detection platform for the detection of PAH in real-life samples such as drinking water and river water where we achieve a detection limit up to 1 nM. We used a CNN classifier to detect the presence of each of the five PAH targets followed by a CNN regressor to quantify each PAH target in the multiplexed sample.

## Materials and Methods

### Materials.

Chloroauric acid (HAuCl_4_), L-ascorbic acid, silver nitrate (AgNO_3_, 99.8%), pyrene (PY), nitro-pyrene (NP), anthracene (ANT), benzo[a]pyrene (BAP), and triphenylene (TP), hydrochloric acid (HCl), trisodium citrate (Na_3_C_6_H_5_O_7_) were purchased from Sigma-Aldrich (USA). The STEM images of the nanostars were collected from Aberration Corrected STEM-Thermo Fisher Titan 80–300. The UV-vis spectra of the nanostars were recorded using a Shimadzu UV-3600i spectrometer with cuvettes of 1 cm path length.

### GNS Synthesis.

Surfactant-free GNS were synthesized by following our previously reported method.^57^ Briefly, 200 μL of 1 M HCl was added to a solution containing 50 mL of 0.25 mM HAuCl_4_ and 15 μL of the citrate capped gold seed solution. Then, 2mL of 3 mM AgNO_3_ and 1 mL of 100 mM ascorbic acid were added to the solution with 5 second time interval. The solution was stirred for 2 minutes. Then, 10 mL 1 mM CTAB solution was added to GNS solution and stirred for 1 hour. The GNS solution was centrifuged at 4000 g for 12 min and dispersed in 10 mL of Milli-Q water so that the final concentration was concentration was five times higher than synthesized nanostars. The final concentration of CTAB capped GNSs was calculated to 2 pmol/L by following our previously reported procedure.^58^ As the GNSs were centrifuged and dispersed in water, we believe that the amount of CTAB in the GNSs solution is negligible.

### Raman Measurements.

The SERS measurement was carried out using a lab built portable system having 785 nm laser source (Rigaku Xantus TM-1 handheld Raman device), a fiber optic probe (InPhotonics RamanProbe), a spectrometer (Princeton Instruments Acton LS 785), and a CCD camera (Princeton Instruments PIXIS: 100BR_eXcelon). The laser power of the Rigaku Xantus TM-1 was set at 200 mW and the CCD camera exposure time was set at 3s. The SERS measurement was standardized using ethanol. The SERS measurement involves a minimal sample preparation in which 300 μL of the gold nanoparticle solution (10 μL stock solution of GNS + 287 μL of Milli-Q-water) were thoroughly mixed with 3 μL of analyte solution in a plastic cup cut from a 1.5 mL centrifuge tube which was covered with aluminum foil to prevent signal interference from the polypropylene well plate.

### Training data simulation for machine learning.

Data for training was calculated as described previously ^54^, with modifications. Briefly, 100 μM solutions of each pollutant: PY, TP, NP, BAP, and ANT were measured separately on the SERS substrate as described above and used as the reference spectra. Five spectra were acquired for each reference. A blank was measured using tap (drinking) water. The data were preprocessed by smoothing in MATLAB using a Savitsky-Golay filter (span of 5, polynomial degree 3), and finally background subtracted by Savitsky-Golay filter (span of 5, polynomial degree 1).

Spectral mixtures were calculated by scaled addition of reference spectra in MATLAB. Data sets for training (n=10,000), validation (n=2,000), and optimization (n=6,000) were calculated using the same procedure. Multiplexed spectra were calculated by scaled addition of reference spectra, where scaling factors were used as output labels for the model. For regression model, an additional term was added to output label denoting a normalization factor used in conversion to concentration. Gaussian noise and random shifts were added to the spectra to simulate experimental conditions.

### Convolutional neural network for classification and regression.

CNN architecture was tuned in Optuna using n=6,000 optimization calculated dataset (Figure S3-S5, S6-S8, and Table S1-S2). The optimized CNN consisted of 3 convolutional layers and 1 dense layer (Figure 4). Slightly different architectures were used for classification and regression tasks. CNN were trained on n=10,000 training set and validated using n=2,000 validation set to prevent overfitting. Both models were evaluated using all four test sets (including drinking and river water samples). For classification, outputs were thresholder based on the geometric mean (√sensitivity*specificity). For regression, continuous predictions were converted to concentration by dividing each contribution label by predicted normalization factor, scaling each by the maximum height of the unnormalized test spectrum and converting to concentration based on the calibration curve for each respective target with a constraint of lowest value =0.

## RESULTS AND DISCUSSION

### Synthesis of GNS.

As an important aspect of the proposed ultrasensitive and highly reproducible SERS sensing platform for the accurate and quantitative detection of PAH, we synthesized highly stable CTAB capped surfactant-free GNS that generate a strong, and uniform SERS signal. Surfactant-free GNS synthesis was selected, which can generate multiple number of spikes in absence of any surfactant.^57^ However, the reported surfactant-free GNS are thermodynamically not stable, and they become spherical shape. Interestingly, it is reported for a surfactant based GNS that at high Ag^+^ concentration, the nanostars morphology is stable, which is probably due to deposition of a thin layer of silver on the spikes and stabilizing the nanostar morphology.^48^ Herein, the concentration of AgNO_3_ was tuned from 30 μM to 120 μM to explore the nanostars morphology and stability, whereas the other parameters (ascorbic acid, HAuCl_4_, HCl, and gold seeds) concentration for GNS growth were fixed. We have achieved three different morphologies of GNS: GNS-1, GNS-2, and GNS-3. In the second step, the surfactant-free GNS were capped with CTAB, which makes the GNS surface more hydrophobic; hence facilitating the adsorption of non-polar compounds like PAH. It is important to note that a minimum amount of CTAB is necessary on the GNS surface to capture the analytes. Excess free CTAB molecules present in the solution can interact with the analytes. As the free CTAB molecules are not close enough to the GNS, it can reduce the overall SERS signal. Therefore, we centrifuged the CTAB capped GNS and tried to remove the supernatant as much as possible to get rid of excess CTAB. Moreover, we observed that CTAB capped GNSs were stable in water for long storage time. However, they are not stable for long term in other solvents such as ethanol, methanol. They aggregated after 2–3 days, when they stored in ethanol.

**Figure 1.**
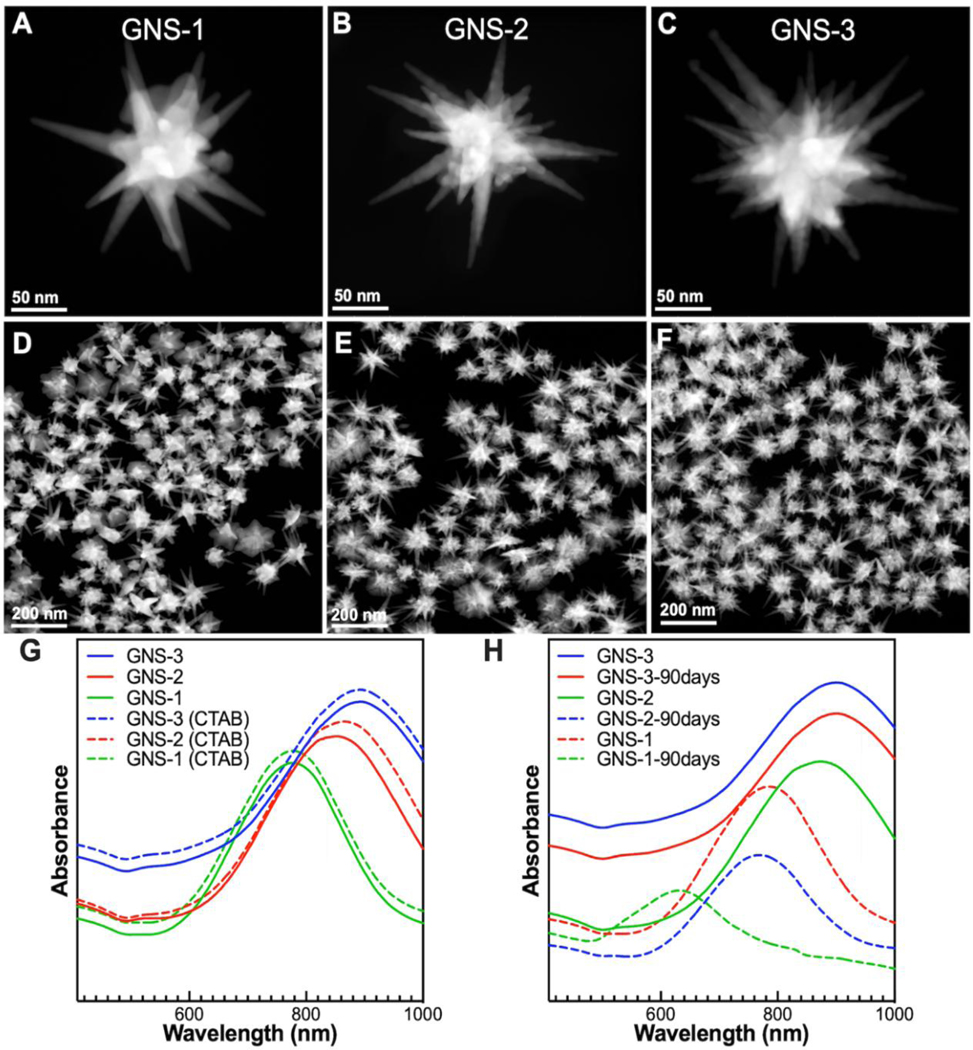
STEM images of GNS-1, GNS- 2, and GNS-3 (A-C). STEM images GNS-1, GNS-2, and GNS-3 with multiple nanostars showing high monodispersity of the syntheses (D-F). UV-vis spectra of surfactant-free and after CTAB coated GNS-1, GNS-2, and GNS-3 showing that the plasmon resonance was red shifted after CTAB capping (G). UV-vis spectra of CTAB capped GNS-1, GNS-2, and GNS-3 after 90 days of synthesis showing that the maximum plasmon resonance for GNS-1, and GNS-2 was blue shifted after 90 days of synthesis, whereas it remained fixed at 902 nm for GNS-3 indicating high stability of GNS-3 (H).

Figure 1A-C displays the typical morphology of GNS-1, GNS-2, and GNS-3, which shows that the spike length and number were increased from GNS-1 to GNS-3 and reaches maximum for GNS-3. STEM images of multiple stars indicate high monodispersity of the syntheses (Figure 1D-F). The UV-vis spectrum shows that the maximum plasmon resonance of GNS-1, GNS-2, and GNS-3 were at 770 nm, 850 nm, and 895 nm, respectively (Figure 1G). The absorbance values plotted in Figure 1G-H represent the relative values to show the spectral shifts of the plasmon bands for different gold nanostars. Some of the absorbance curves are moved upward in the graph to prevent overlap of the spectra in order to minimize confusion. It is important to note that there was around 7 nm red shift after CTAB capping of GNS (Figure 1G). The synthesis of the surfactant-free gold nanostars requires very careful control of the parameters including precise amount of ascorbic acid, AgNO_3_, seeds, and HAuCl_4_. Also, the timing of the addition of these chemical components (ascorbic acid and AgNO_3_) is also very critical and must be well controlled precisely. We have carefully studied these experimental factors and have now achieved good reproducibility of the nanostars synthesis. The variation of the plasmon maximum band of nanostars is within only 5 nm (Figure S1d).

We further investigated the stability of the GNS over 90 days. The blue-shifting of the plasmon resonance of GNS-1 in the UV-vis spectra (Figure 1H) was further confirmed that the morphology of GNS-1 was changed (Figure S1a-1b). Interestingly, there was no change in morphology for GNS-3 (Figure S1c).The high stability of GNS-3 was attributed to the underpotential deposition of silver layer on the spikes which prevents the movement of highly energetic gold atoms present at the tips of the spikes.^48^ On the other hand, the spike length for GNS-1, and GNS-2 was reduced with time. Figure S1 shows that the morphology of GNS-1 was almost spherical after 90 days of synthesis, which is probably due to diffusion of gold atoms at the tips of nanostars diffused towards the core the nanostars. We believed that the amount of underpotential deposition of silver layer on GNS-1 and GNS-2 is negligible compared to GNS-3. Therefore, the highly energetic gold atoms at the tips moved towards favorable spherical core of the nanostars.

### SERS measurement of PAH.

**Figure 2.**
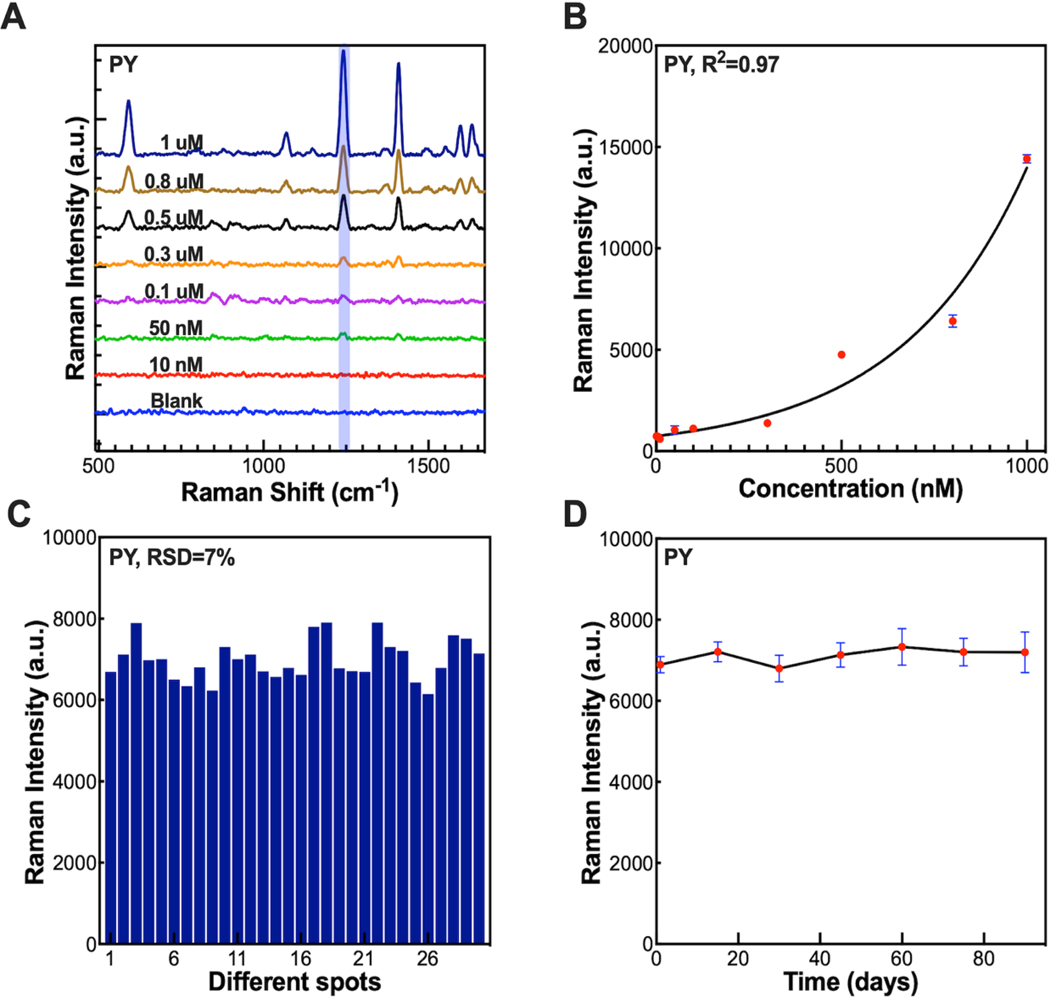
SERS spectra of PY (A) with the concentration ranging from 1 μM to 10 nM. The calibration curve for the SERS peak intensity of PY at 1241 cm^−1^ from 1 μM to 10 nM concentrations (B). Reproducibility of the SERS platform (C). Stability of the SERS platform (D).

We have used the highly stable CTAB capped GNS-3 for the SERS detection of PAH. Figure 2A shows the SERS spectra of PY at different concentration ranging from 1μM to 10 nM. The main characteristic SERS band for PY is located at 1241 cm^−1^, which is assigned to the ring C=C stretching vibration.^59^ Figure 2B displays the calibration curve of PY, which shows a polynomial curve fit between the concentration of PY and the SERS signal intensity at 1241 cm^−1^. The LOD was estimated to be 10 nM for PY with a good signal-to-noise ratio (S/N = 3.5). We further investigated the SERS signal reproducibility and stability. Figure 2C shows the SERS signal intensity of PY at 1241 cm^−1^ of 30 different samples where the relative standard deviation (RSD) value was 7 % indicating good reproducibility. Figure 1D displays the SERS intensity of PY at 1241 cm^−1^ over 90 days of synthesis underscoring the high stability of our SERS platform. We further investigated batch-to-batch reproducibility of GNS-3. The relative standard deviation (RSD) of the SERS signals with GNS-3 from five batches is 4.8%, indicating good batch-to-batch reproducibility of our SERS platform (Figure S2).

**Figure 3.**
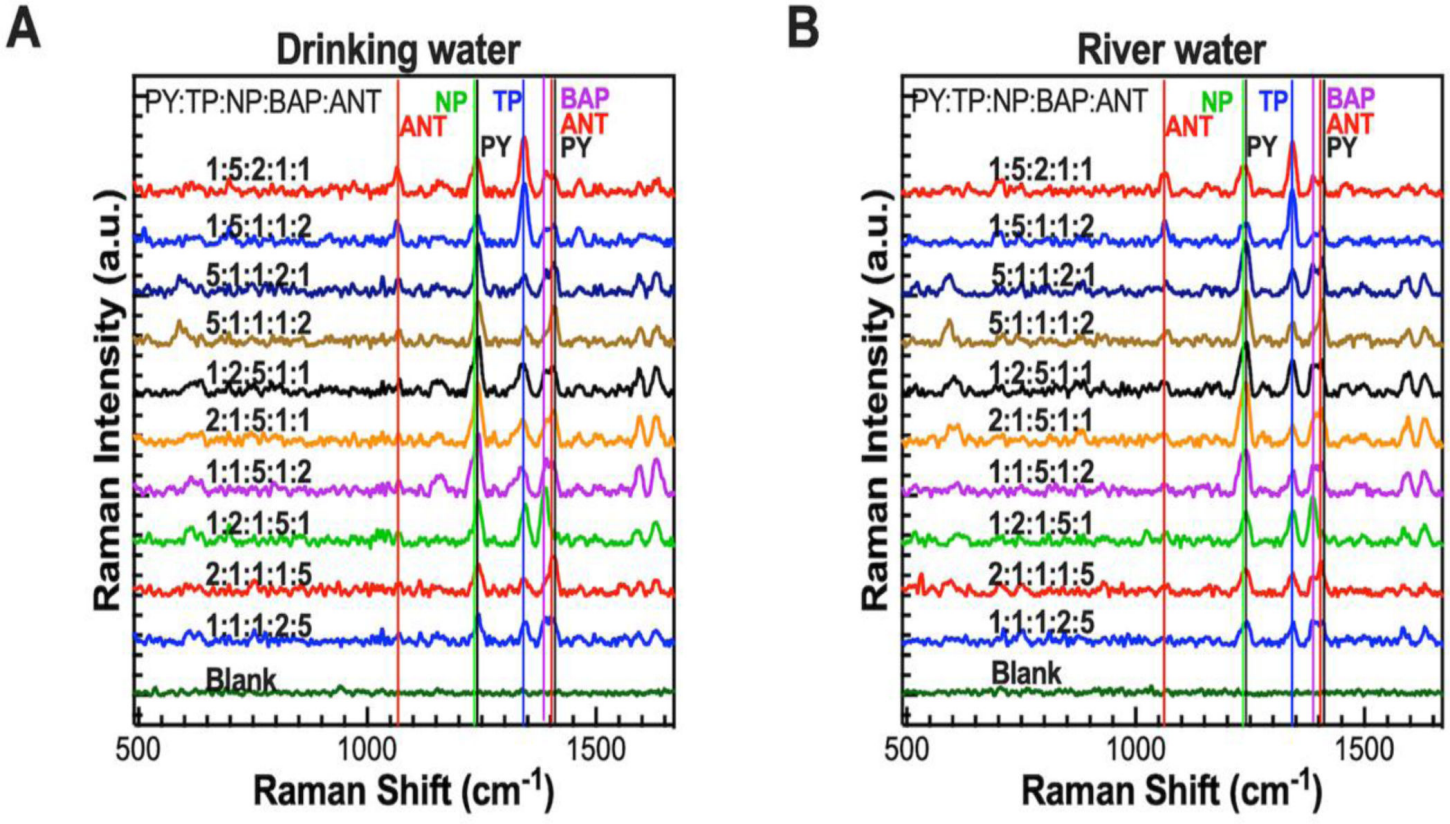
Multiplex SERS spectra of PY, TP, NP, BAP, and ANT spiked in drinking water (A), and river water (B) at different ratios.

Based on our satisfactory SERS analysis of PY using GNS-3, we explored the other four PAH. Figure S3 displays the SERS spectra and calibration curve of NP, ANT, BAP, and TP, and a polynomial curve fit between the concentration of the PAH and SERS signal intensity. The LOD was estimated to be 1 nM, 10 nM, 10 nM, 10 nM for TP, NP, BAP, ANT, respectively, with a good signal-to-noise ratio (S/N = 3.5) (Figure S4). We further investigated multiplex detection of PAH in complex real-life samples. In this study, we spiked the mixture of all five PAH in drinking water, and river water where the ratio of the PAH is 5:2:1:1:1 and the total concentration of PAHs was kept constant. For example, when the ratio of PY, TP, NP, BAP, and ANT were 5:2:1:1:1, the concentration of PY, TP, NP, BAP, and ANT were 0.5 μM, 0.2 μM, 0.1 μM, 0.1 μM, and 0.1 μM, respectively. As seen from Figure 3, the SERS spectra of the mixture of five PAH in the mixture displays some overlap especially for PY, NP, and ANT as the main characteristic peaks around 1240, and 1400 cm^−1^. We have further investigated the SERS data using machine learning algorithm to determine the PAH concentrations more accurately and precisely.

### Machine learning algorithms for multiplex detection of PAH.

In the past decades, there has been substantially advanced progression in nanotechnology for fast, in-field detection of analyte molecules. However, due to complexity of multi-analyte spectra, irregular signal noise, and signal shifts between experiments, analysis of SERS spectra from real-world samples by conventional techniques such as spectral decomposition can result in poor accuracy and reliability, restricting SERS application for commercialization. Recent emerging need of artificial intelligence (AI) models for spectral analysis can extract signal information more accurately in complex environments. Compared to other AI models, CNNs in particular show good performance in both classification^53^ and regression analysis ^54^ of SERS spectra.

**Figure 4.**
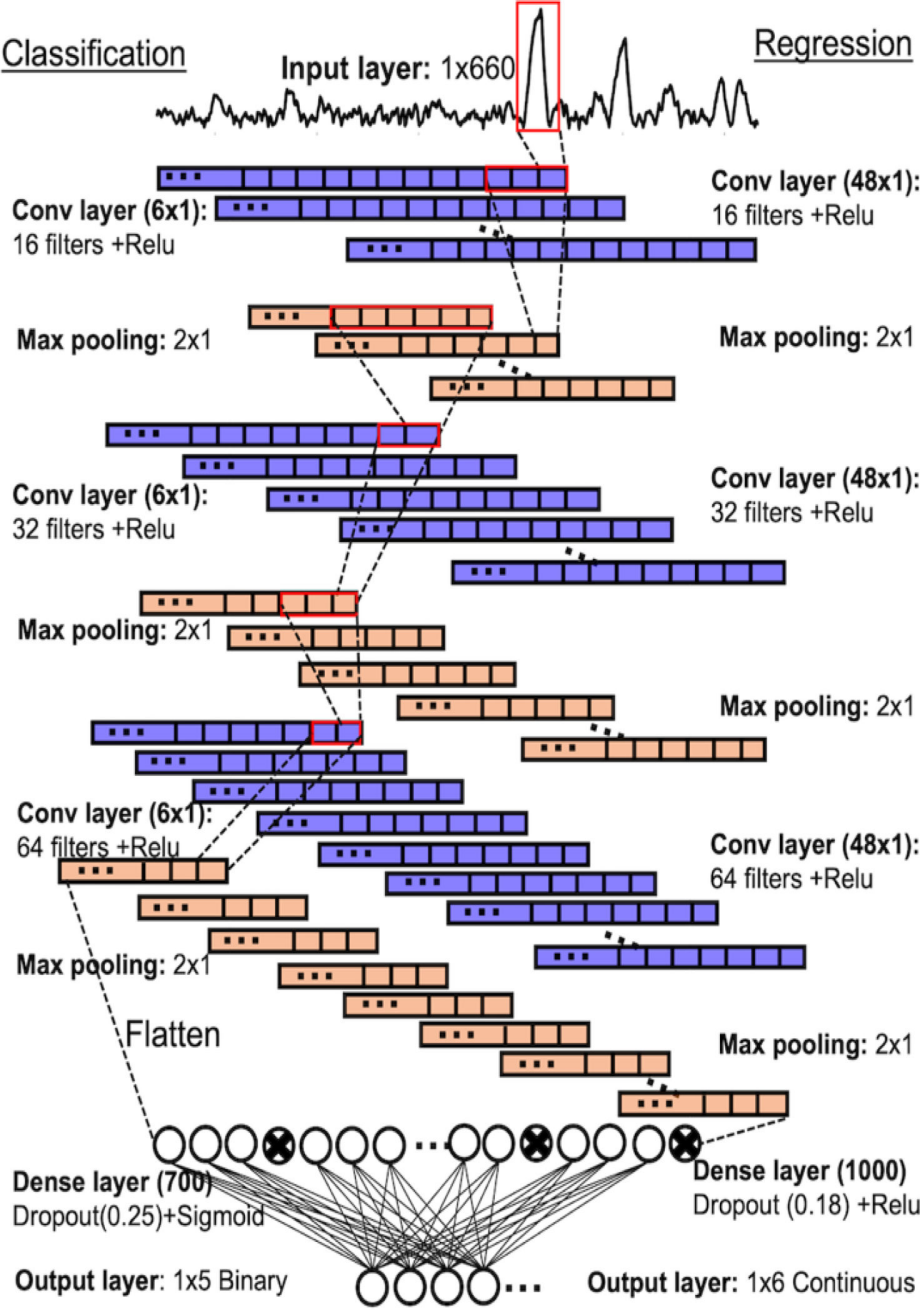
CNN architectures for both classification (left) and regression (right) models.

To improve the accuracy of our SERS-based PAH detection method, we used two 1D convolutional neural networks (CNNs) with architectures shown in Figure 4 (left) for multiclass classification: identifying pollutants and Figure 4 (right) for regression: predicting pollutant concentrations. Four different test sets were acquired (n=39 total). Two test sets consisted of SERS spectra obtained from spiked drinking (tap) water and two consisted of SERS spectra obtained from spiked river water. For each water type, one set contained spectra from samples containing all 5 pollutants, combined in different concentrations, while the other set contained spectra from samples containing 2 of 5 pollutants, combined in equal concentrations. CNN models require extensive data for training, which is costly to acquire experimentally. Our previous work showed training CNN models using calculated data yielded good performance. Therefore, mixture SERS spectra were calculated for training and validation using a modified procedure from. ^54^

Briefly, a library of reference spectra was obtained for each pollutant, references were scaled and added, followed by addition of random noise and shifts. The modified procedure randomly set target contributions to zero with 20% probability and set a minimum contribution of 1% of total signal. Data simulation procedure is detailed in SI. Each spectrum had two corresponding labels, each made up of a string of numbers. CNN classifier used a binary string label of 5 numbers denoting presence or absence of each target, and CNN regressor used a continuous label of 6 numbers quantifying contribution of each target in the normalized spectrum plus an extra normalization term to correct for overlapping peaks in reference spectra. First, the CNN architecture was optimized using Optuna for both models, see details in Table S1-S2, Figure S5-S10. Then, both CNNs were trained on a calculated dataset of n=10,000 and validated on dataset of n=2,000. The threshold for each target in the CNN classification model was determined based on the highest geometric mean (√sensitivity*specificity) from the validation set. Finally, the trained CNNs were used to evaluate the four different test sets.

**Figure 5.**
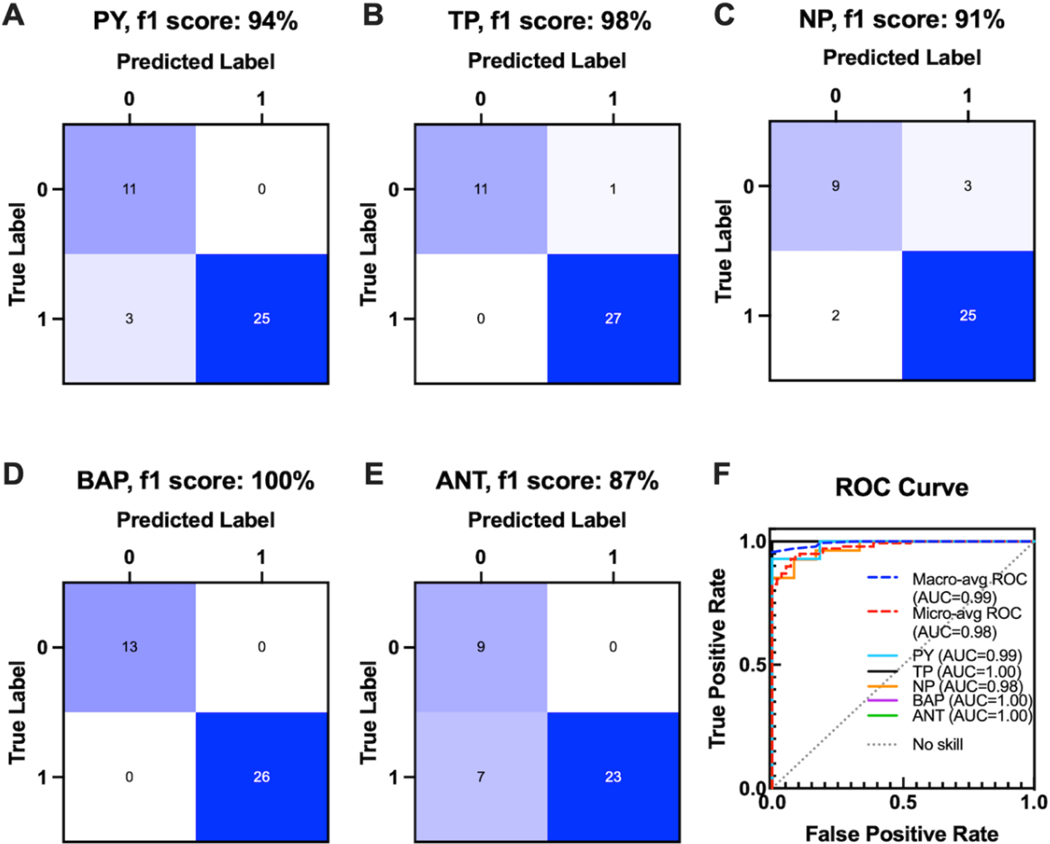
Binary confusion matrices for each pollutant: PY (A), TP (B), NP (C), BAP (D), and ANT (E). Specificity, sensitivity, negative predictive value, precision, and f1 score were displayed for each pollutant. ROC curve (F) for each of the five pollutants (PY, TP, NP, VAP, and ANT) and micro and macro averages compared to no skill (grey dotted line). Corresponding AUC for each is displayed in legend.

Performance of the classification model was evaluated by their precision, specificity, sensitivity, negative predictive value, and f1 score (Table 1). F1 score was chosen over accuracy because the combined test sets contain more positive (~70%) than negative samples (~30%), and f1 is a better evaluation metric in unbalanced datasets. The model performed well with a micro average of 97% precision, 94% f1 score, and accuracy of 90% (Table S4). ANT had the lowest f1 score of 87% while BAP had perfect precision and f1 score. To further evaluate model performance, binary confusion matrices were plotted for each target (Figure 5A-E). Prediction accuracy could be related to the complexity of the SERS spectrum. The SERS spectrum of ANT, the worst predicted pollutant, has the least number of spectral peaks (three), and the SERS spectrum of BAP, the best predicted pollutant, has the most spectral peaks (eight). The ANT SERS spectrum also had the lowest intensities for the same concentrations (Figure 3C), which could also contribute to its low recall. NP had the lowest precision due to its relatively higher number of false positives. Although the NP SERS spectrum had more peaks than ANT, it did not have any unique peaks. Since all its peaks overlapped with one or more peaks of the other targets, false positives were more likely, resulting in lower precision. See Table S4-S5 for a breakdown of f1 scores by target and different test sets. Thus far, the performance evaluation metrics considered have been dependent on thresholding.

The receiver operating characteristic (ROC) curve is an evaluation metric that shows the performance of a classification model at all thresholds. The area under the curve (AUC) of the ROC curve measures performance independent of threshold. A completely random predictor would have an ROC-AUC of 0.5 while a perfect predictor would have an ROC-AUC of 1. ROC was plotted for each pollutant target, micro, and macro averages (Figure 5F). The model achieved an AUC micro average of 0.98 and macro average of 0.99. Overall, type II errors (false negatives) were the most common.

Next, a CNN regression model was used to predict the height contribution of each target pollutant SERS spectrum to the test spectrum. Then the CNN prediction and maximum spectral values are used to quantify pollutant concentrations by converting height contributions to concentrations using the calibration curves of each separate target (Figures 2, and S3). A predicted spectrum was calculated by scaled addition of reference spectra based on the predicted labels. The model performance was evaluated using two metrics: 1) ${\text{RMSE}}_{spectrum}$ (eqn. 1), which refers to the root-mean-squared-error between the spectral values of the predicted and actual spectrum; 2) ${\text{RMSE}}_{conc}$ (eqn. 2), which refers to the root-mean-squared-error between the predicted and actual concentrations in μM.


(1) $$RMSE_{spectrum}=\sqrt[{2}]{\frac{1}{n*w}\sum_{i=1}^{n}\sum_{k=1}^{w}{\left(\hat{y}spectrum_{i,k}-Yspectrum_{i,k}\right)}^{2}}$$

(2) $$RMSE_{conc}=\sqrt[{2}]{\frac{1}{n*m}\sum_{i=1}^{n}\sum_{j=1}^{m}{\left(\hat{y}conc_{i,j}-Yconc_{i,j}\right)}^{2}}$$

Equations (1) Root mean squared error between actual and predicted spectral points: $\hat{\text{y}}spectrum_{\text{i},\text{k}}$ and $\text{Y}spectrum_{\text{i},\text{k}}$ denote the predicted and ground truth spectral values respectively, where spectral values correspond to each value in the measured SERS spectrum, i denotes indexing of sample number from 1 to n and k denotes indexing of number of spectral values for each sample from 1 to w=660. Equation (2) Root mean squared error between actual and predicted concentrations: $\hat{\text{y}}conc_{\text{i},\text{j}}$ and $\text{Y}conc_{\text{i},\text{j}}$ denote the predicted and ground truth concentrations respectively, i denotes indexing of sample number from 1 to n and j denotes indexing of number of targets from 1 to m=5.

**Figure 6.**
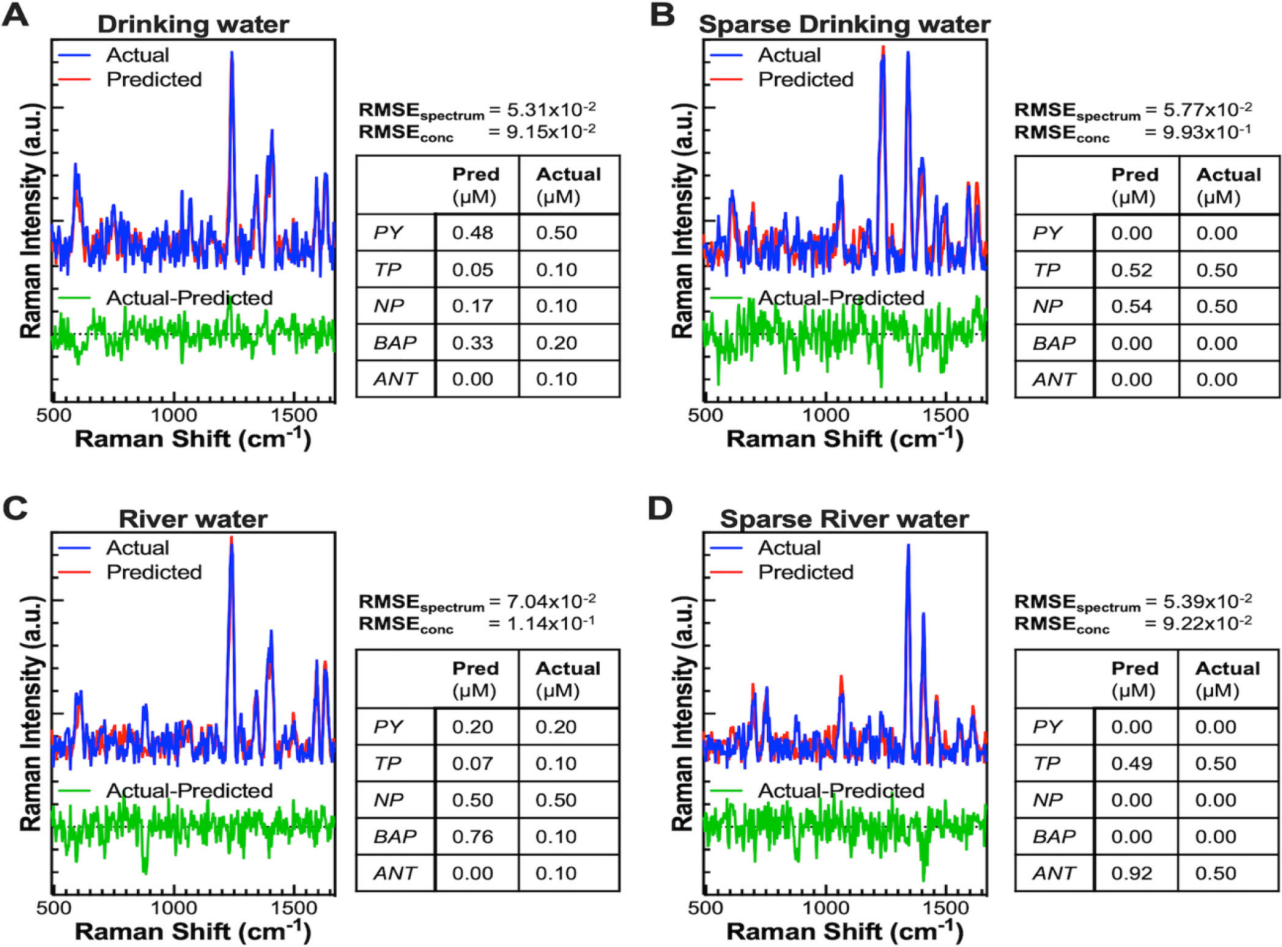
Example spectrum and CNN regression model performance from the drinking water test set (A), sparse drinking water test set (B), river water test set (C), and sparse river water test set (D). For (A-D), Top: predicted (red) and actual spectrum (blue) plotted together. Bottom: difference between actual and predicted spectrum. Right: RMSE_spectrum_ and RMSE_conc_ for the respective test set. Table shows predicted and actual concentrations in μM for each target pollutant in the example spectrum plotted to the left.

The regression model performed well with a combined ${\text{RMSE}}_{spectrum}$ of 5.92×10^−2^ and ${\text{RMSE}}_{conc}$ of 1.07×10^−1^ (μM). Model performance for each test set is shown in Figure 6A-D. ${\text{RMSE}}_{conc}$ and ${\text{RMSE}}_{spectrum}$ metrics generally agree except in a few cases. The best ${\text{RMSE}}_{spectrum}$ and ${\text{RMSE}}_{conc}$ were both in the drinking water test set while the worst ${\text{RMSE}}_{spectrum}$ occurred in the river water test set and the worst ${\text{RMSE}}_{conc}$ occurred in the river water sparse test set. This difference could be because ${\text{RMSE}}_{spectrum}$ is affected by spectral noise, or because of background differences in river water vs. drinking water, with which reference spectra were taken. Example of actual vs. predicted spectra and concentrations in Figure 6A-D (left) show the close match between actual and predicted spectra.

### Conclusion

In this work, we have employed GNS with sharp spike and maximum number of spikes as a sensitive SERS sensor for detection of PAH. The GNS was synthesized by a simple surfactant-free seed-mediated synthesis method, and they were capped with CTAB surfactant to trap the PAH molecules inside the CTAB bilayers. The SERS platform with GNS-3 exhibit excellent sensitivity and reproducibility. AI based on CNN algorithms can be used to predict PAH pollutant concentrations from multiplexed, SERS spectra obtained by our sensor from spiked drinking water and river water samples. Using a CNN classification model, we have achieved a high prediction accuracy of 90% in the nanomolar detection range and f1 score of 94%; using a CNN regression model, we have achieved a RMSE_conc_=1.07×10^−1^ μM. The present machine learning approach can be applied for the detection of a variety of organic non-polar organic pollutants. It can also be used for other applications such as analysis of SERS spectra of biomarkers for medical diagnostics.

## Supplementary Material

SI

Additional experimental details, calibration curves, CNN optimization, and CNN results (DOC).
