# Supplementary material for: Multiplex SERS Detection of Polycyclic Aromatic Hydrocarbon (PAH) Pollutants in Water Samples Using Gold Nanostars and Machine Learning Analysis
Source: Analyst. Author manuscript; Available in PMC 2026 Jan 6. (PMC12768947; doi:10.1039/d3an00636k)
Supplement: SI [file NIHMS1929907-supplement-SI.docx]

Multiplex SERS Detection of Polycyclic Aromatic Hydrocarbon (PAH) Pollutants in Water Samples Using Gold Nanostars and Machine Learning Analysis

*Supriya Atta, ^a, b^Joy Li, ^a, b^ Tuan Vo-Dinh ^a,^ ^b, c‡^*

^a^ Fitzpatrick Institute for Photonics, ^b^ Department of Biomedical Engineering, ^c^ Department of Chemistry, Duke University, Durham, NC 27708, USA.

**CONVOLUTIONAL NEURAL NETWORK SECTION**

Reference Acquisition for Data Augmentation- 100 µM solutions of each pollutant: PY, TP, NP, BAP, and ANT were measured separately on the SERS substrate as described above and used as the reference spectra. Five spectra were acquired for each reference. A blank was measured using tap (drinking) water.

**Data Preprocessing**

The 488-1666 cm^-1^ range was used for analysis due to its rich SERS content. The raw reference spectra were blank-subtracted by scaled subtraction of blank spectra from sample spectra, smoothed in MATLAB using a Savitsky-Golay filter (span of 5, polynomial degree 3), and finally background subtracted by Savitsky-Golay filter (span of 5, polynomial degree 1).

**Training, Validation, and Optimization Set Generation**

Spectral mixtures were simulated by scaled addition of reference spectra in MATLAB. Data sets for training (n=10,000), validation (n=2,000), and optimization (n=6,000) were simulated using the same procedure. During data augmentation, one of the five repeats of each reference spectrum is randomly selected to simulate the mixture. Firstly, for each of the 5 distinct references, the background was separated from raw spectrum by subtracting the preprocessed spectrum (blank-subtracted, background-subtracted) from the raw spectrum. Next, the raw spectrum was normalized by the maximum intensity of the preprocessed spectrum. Then, the normalized, raw spectra were multiplied by the simulated scaling factor (label). Finally, the spectra were randomly shifted to the left or right in the range [0, 4]. Gaussian noise was added at random power in the range [1x10^-8^, 1x10^-3^], resulting in a simulated raw spectrum. Finally, the raw spectrum was processed by scaled blank subtraction, background subtraction using Savitsky-Golay as described above, and normalized by the maximum intensity of processed spectrum so all spectral points are in the range [0, 1].

**Label Generation**

Label for each training spectrum consisted of a vector of five scaling factors, corresponding to each reference spectrum. Each label vector is summed to 1. Labels were generated by creating an evenly spaced calibration curve of one reference in the range [5x10^-2^, (1-1.5x10^-1^)]. Concentrations of all other references generated randomly such that sum of all labels equaled 1. Then, all labels in the vector were set to zero with an independent probability of 20%. Any randomly generated label less than the minimum of 5x10^-2^ was set to zero and the label vector was renormalized to sum to 1. This was repeated until a calibration curve was simulated for each distinct reference. The resulting labels were used as the first 5 labels for regression models. A 6^th^ label was added to each spectrum to denote a normalization factor. This last label was defined as the maximum height of the simulated spectrum based on generated labels and normalized references. The normalization factor is related to the amount of spectral overlap between the reference spectra. To obtain binary labels for classification models, all non-zero labels (first 5) were set to 1.

**Stability of GNS**


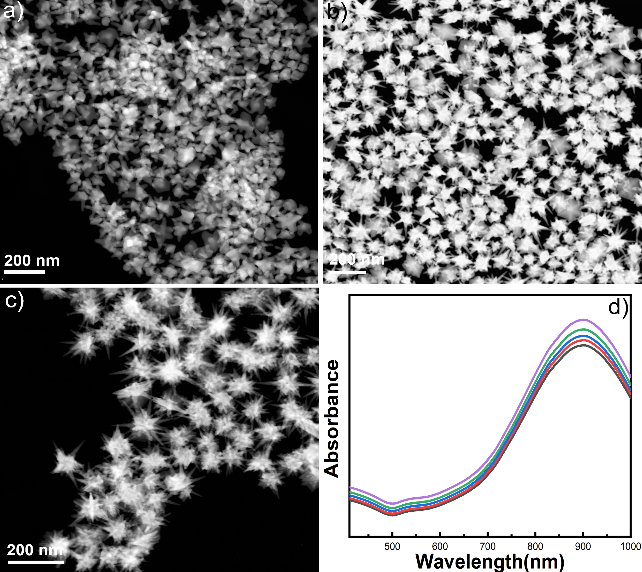


**Figure S1.** STEM images of GNS-1 (a) GNS-2 (b), and GNS-3 (c) after 90 days of synthesis. (d) UV-Vis absorbance spectra of five different batches of GNS-3.


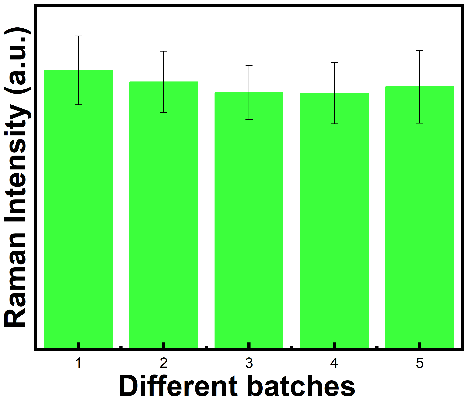


**Figure S2.** SERS peak intensity of PY at 1241 cm^-1^ at 1 µM concentration with five different batches of GNS-3.


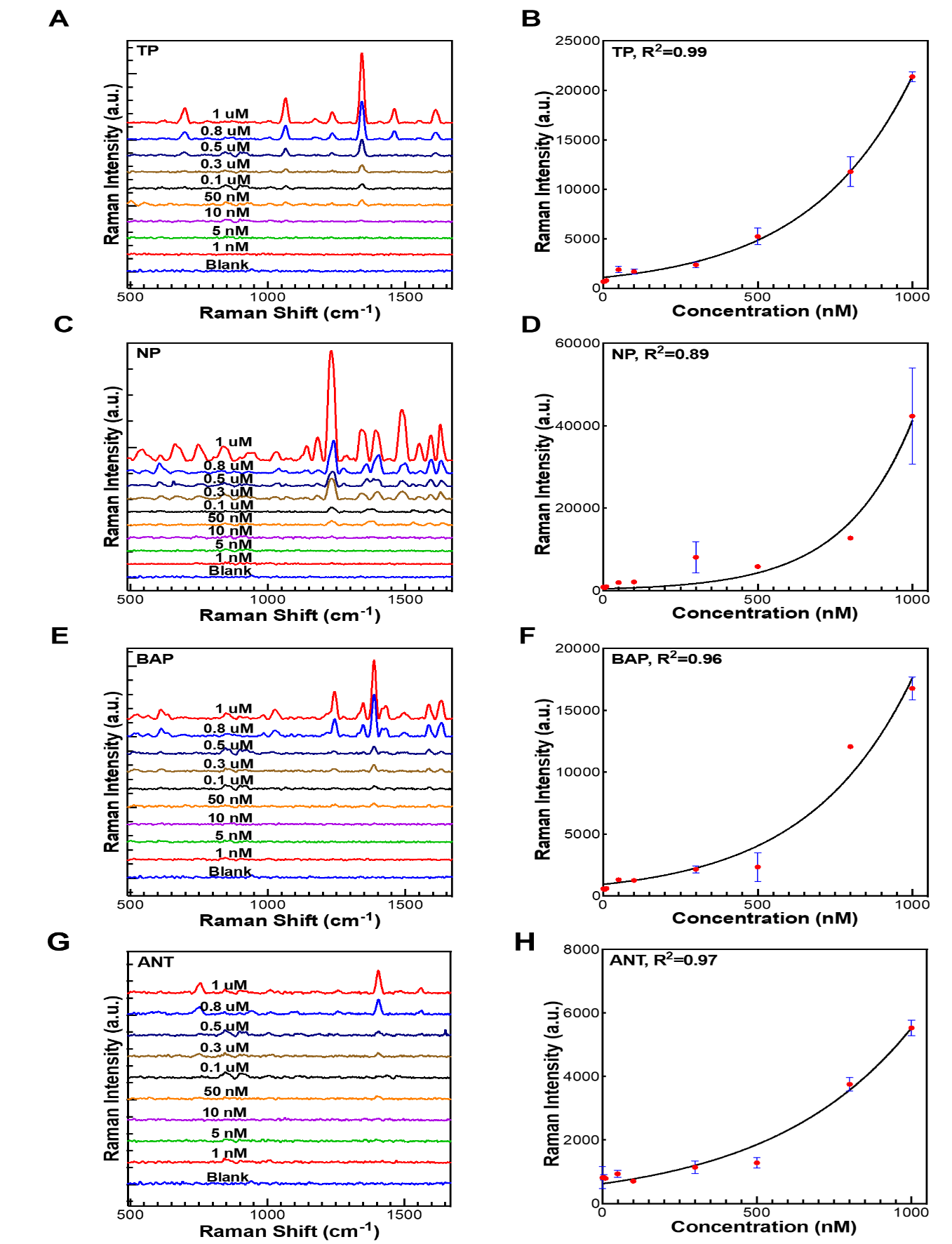


**Figure S3.** SERS spectra of BAP (A) with the concentration ranging from 1 uM to 10 nM. The calibration curve for the SERS peak intensity of BAP at 1386 cm^-1^ from 1 uM to 10 nM concentrations (B). The SERS spectra and calibration curve of NP (C, D) TP (E, F). The SERS spectra of ANT (G) with the concentration ranging from 1 uM to 10 nM. The calibration curve for the SERS peak intensity of ANT at 1405 cm^-1^ from 1 uM to 10 nM concentrations (H).


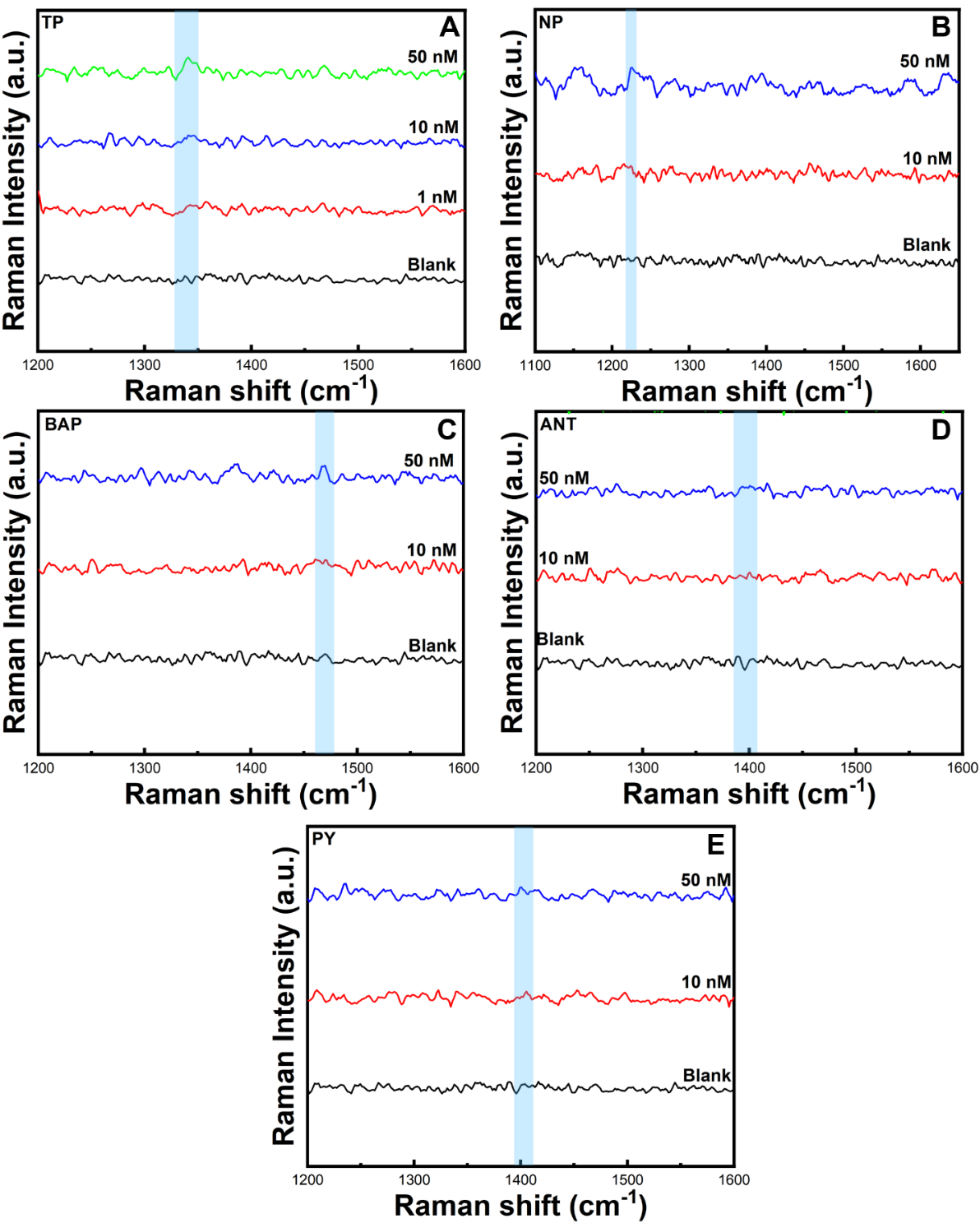


**Figure S4.** SERS spectra of TP, NP, BAP, ANT, and PY with the concentration ranging from 50 nM to 10 nM (A-E).

**CNN Architecture Optimization for Classification**

CNN architecture was tuned using 5-fold cross validation in Optuna using 40 trials. A n=6,000 dataset was generated using the above technique with corresponding binary labels. Static parameters (Table S1) tuned hyperparameters (Table S2), and optimization visualization (Fig. S1-S3) are shown below. CNN hyperparameters with the lowest RMSE was used to evaluate the test set.

Table S1. CNN static parameters used during CNN optimization for classification and regression models.

| **Static Parameter** |  |
| --- | --- |
| Max Pooling (after conv layer) | Stride [1 x 2] |
| Activation Function | ReLU (conv layers)  Sigmoid (dense layer) |
| Dropout | 0.25 |
| Optimizer | Adam Optimizer |
| Batch Size | 3000 |

**Table S2.** Hyperparameters, ranges, and distributions used in tuning of CNN for classification and regression models using Optuna for n=40 trials. RMSE of best PLSR trial.

|  |  |  |  |  |
| --- | --- | --- | --- | --- |
| **Hyperparameter** | **Variable type** | **Tuning distribution** | **Range/Options** | **Parameter of best trial** |
| Kernel Size | categorical | uniform | 6, 12, 24, 48 | 6 |
| No. Dense nodes | categorical | uniform | 300, 500, 700, 1000 | 700 |
| Dropout rate | continuous | log uniform | [5x10^-2^, 3x10^-1^] | 2.5x10^-1^ |
| Learning rate | continuous | log uniform | [5x10^-4^, 2x10^-3^] | 1.24x10^-3^ |
| Best RMSE score |  | 1.88x10^-2^ |  |  |


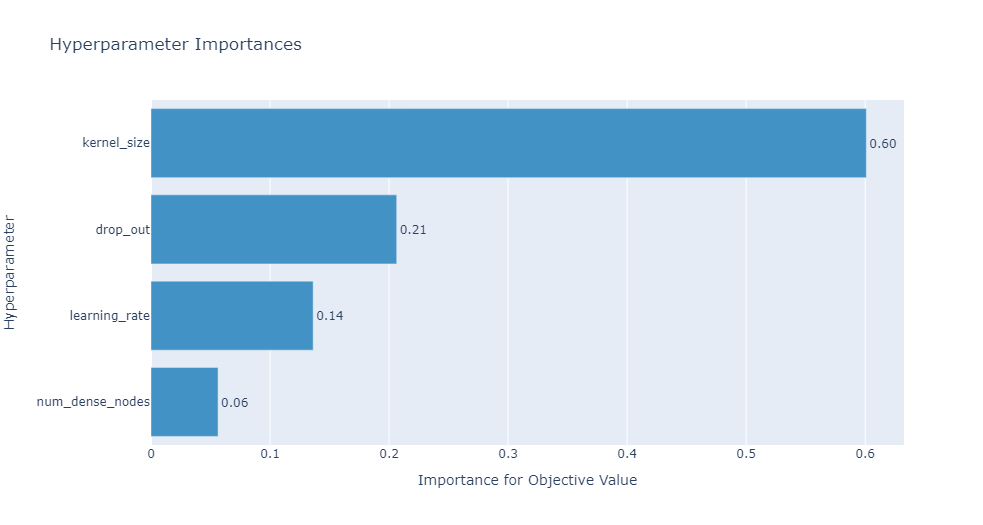


Figure S5. Visualization of hyperparameter importance during optimization of CNN classification model.


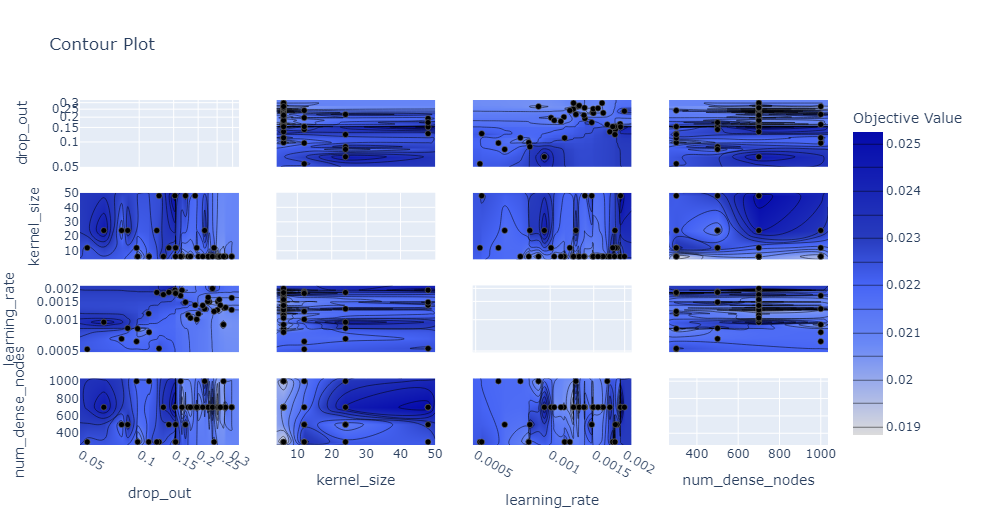


**Figure S6.** Contour plots showing relationships between parameters during CNN classification model optimization.


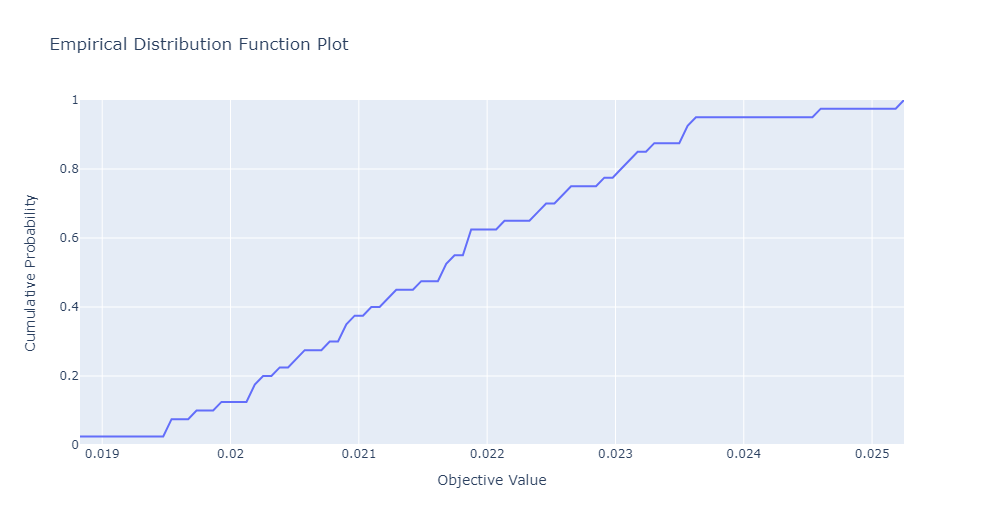


**Figure S7.** Visualization of the objective value empirical distribution function (EDF) of the CNN classification model optimization study.

**CNN Classification Model**

The 1D-CNN was built using TensorFlow in Python and is comprised of an input layer, four 1D convolutional layers, separated by three max pooling layers, followed by a fully connected dense layer with dropout, and an output layer (Fig. S3). The preprocessed SERS spectra is fed to the input layer, which passes to the first convolutional layer comprised of 16 kernels of size 6. A convolutional layer moves a kernel over the spectrum with a stride of 1 and outputs a feature map which is fed to a ReLU nonlinear activation function. A max pooling layer with stride 2 reduces dimensionality of the previous layer, decreasing risk of overfitting, and computational burden. After four convolutional and four max pooling layers, the data are flattened before being fed into the dense layer with a dropout rate of 25%. Dropout is a regularization technique which randomly omits certain nodes during training (at a set probability), which reduces chances of overfitting. A sigmoid activation function is used on the dense layer. Finally, the output layer produces the network’s predictions, consisting of 5 labels between 0 and 1, for multiclass, binary classification.

The Adam optimizer in TensorFlow was used with a learning rate of 1x10^-3^ to compile the CNN with loss as accuracy.

**Thresholding**

The optimal threshold was found separately for each target based on the model predictions on the validation set. Each target threshold was set as the threshold with the highest geometric mean (√sensitivity*specificity). To map the output labels to a binary classification, the prediction labels were thresholder at a 1.71x10^-2^ for PY, 1.81x10^-2^ for TP, 6.15x10^-1^ for NP, 8.42x10^-1^ for BAP, and 9.43 x10^-2^ for ANT.

Table S3. CNN architecture and parameters used for evaluation of test set.

| Type | Kernal Size | No. Kernals | Activation f. | Stride | No. Params |
| --- | --- | --- | --- | --- | --- |
| Conv | 6 | 16 | ReLU | 1 | 112 |
| Max-pooling | 2 | -- | -- | 2 | 0 |
| Conv | 6 | 32 | ReLU | 1 | 3104 |
| Max-pooling | 2 | -- | -- | 2 | 0 |
| Conv | 6 | 64 | ReLU | 1 | 12352 |
| Max-pooling | 2 | -- | -- | 2 | 0 |
| Conv | 6 | 128 | ReLU | 1 | 49280 |
| Max-pooling | 2 | -- | -- | 2 | 0 |
| Flatten | -- | -- | -- | -- | 0 |
| Fully connected | -- | -- | ReLU | -- | 3674300 |
| Dropout | -- | -- | -- | -- | 0 |
| Fully connected | -- | -- | Sigmoid | -- | 3505 |

Model Evaluation

Model performance was evaluated using precision, recall, and F1 score. F1 score is better at evaluating performance of unbalanced datasets. Since the test set contains more than double the number of positive samples to negative samples, F1 is a better evaluator of performance than accuracy. The binary confusion matrices and ROC curve was plotted and AUC was calculated for each pollutant as well as the micro and macro averages.

Table S4. Performance evaluation of CNN classification model based on target including precision, recall, and f1 score.

|  | *Sensitivity (%)* | *Specificity (%)* | *Negative predictive value (%)* | *Precision (%)* | *Recall (%)* | *F1 score (%)* |
| --- | --- | --- | --- | --- | --- | --- |
| PY | 89.2 | 100 | 78.5 | 100 | 89 | 94 |
| TP | 100 | 91.7 | 100 | 96 | 100 | 98 |
| NP | 92.6 | 75 | 81.8 | 89 | 93 | 91 |
| BAP | 89 | 100 | 100 | 100 | 100 | 100 |
| ANT | 76.7 | 100 | 56.3 | 100 | 77 | 87 |
| Macro average | n/a | n/a | n/a | 97 | 92 | 94 |
| Micro average | n/a | n/a | n/a | 97 | 91 | 94 |

|  | *F1 score (%)* | | | |
| --- | --- | --- | --- | --- |
|  | *Drinking water test Set* | *Drinking water sparse test set* | *River water test set* | *River water sparse test set* |
| PY | 82 | 100 | 89 | 100 |
| TP | 100 | 89 | 100 | 100 |
| NP | 89 | 100 | 89 | 86 |
| BAP | 100 | 100 | 100 | 100 |
| ANT | 75 | 100 | 46 | 89 |
| Macro average | 89 | 98 | 85 | 95 |
| Micro average | 90 | 98 | 88 | 94 |

**Table S5.** F1 score of CNN classification model for each of the five targets vs each of the four different datasets.

CNN Architecture Optimization for Regression

CNN architecture was tuned using 5-fold cross validation in Optuna using 40 trials. A n=6,000 dataset was generated using the above technique with corresponding continuous labels. Static parameters (Table S1), tuned hyperparameters (Table S2), and optimization visualization (Fig. S4-S6) are shown below. CNN hyperparameters with the lowest RMSE was used to evaluate the test set.


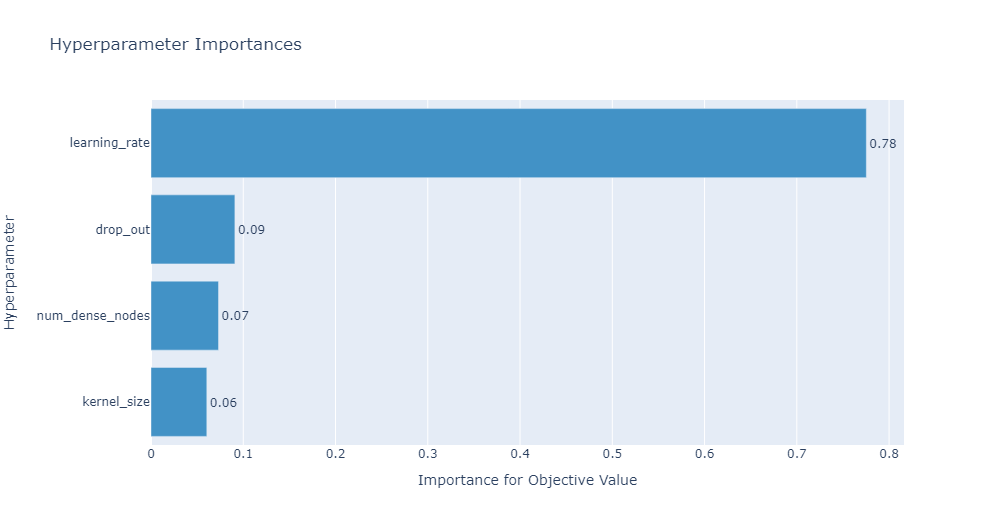


Figure S8. Visualization of hyperparameter importance during optimization of CNN regression.


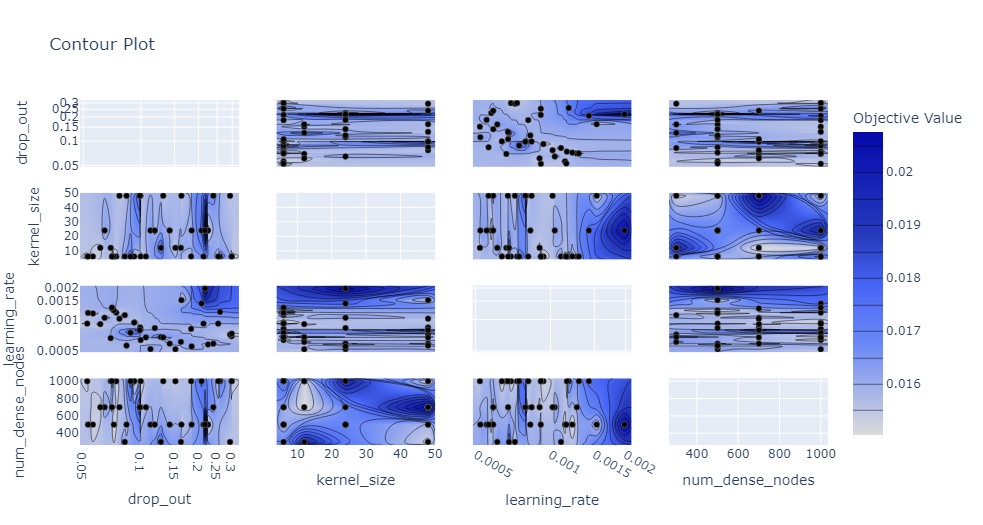


**Figure S9.** Contour plots showing relationships between parameters during CNN regression model optimization.


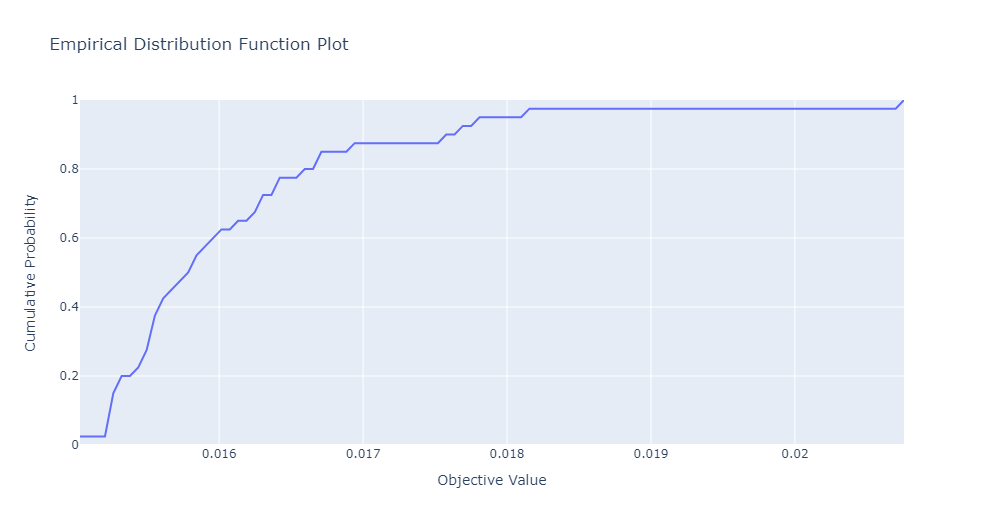


Figure S10. Visualization of the objective value empirical distribution function (EDF) of the CNN classification model optimization study.

CNN Regression Model

The 1D-CNN was built using TensorFlow in Python with similar architecture to classification model. Differences in regression model is the convolutional kernel size of 48, dropout rate of 0.2, and learning rate of 5x10^-4^. Relu was used for activation in all layers including the dense laer. activation function is used on the dense layer. Finally, the output layer produces the network’s predictions, consisting of 6 labels. The first 5 labels consist of continuous numbers between 0 and 1 denoting the fractional contribution of each target, for multiclass regression. The last label consists of a continuous normalization factor. This factor was defined as the maximum height of the simulated reference spectrum before renormalization.

The Adam optimizer in TensorFlow was used to compile the CNN with loss as mean squared error between predicted and true labels.

Table S6. CNN architecture and parameters used for evaluation of test set.

| Type | Kernal Size | No. Kernals | Activation f. | Stride | No. Params |
| --- | --- | --- | --- | --- | --- |
| Conv | 6 | 16 | ReLU | 1 | 784 |
| Max-pooling | 2 | -- | -- | 2 | 0 |
| Conv | 6 | 32 | ReLU | 1 | 24708 |
| Max-pooling | 2 | -- | -- | 2 | 0 |
| Conv | 6 | 64 | ReLU | 1 | 98368 |
| Max-pooling | 2 | -- | -- | 2 | 0 |
| Conv | 6 | 128 | ReLU | 1 | 393344 |
| Max-pooling | 2 | -- | -- | 2 | 0 |
| Flatten | -- | -- | -- | -- | 0 |
| Fully connected | -- | -- | ReLU | -- | 5249000 |
| Dropout | -- | -- | -- | -- | 0 |
| Fully connected | -- | -- | ReLu | -- | 5005 |

**CNN Regression Performance Evaluation**

Predicted normalized spectrum was simulated from predicted labels by a process similar to data simulation. Averaged reference spectra are scaled by predicted label and combined to form the predicted spectrum. The predicted and actual spectra for each test set were plotted (Fig. S7-S10). RMSE*_spectrum_* and RMSE*_conc_* were calculated for each test set and combined test sets.

**Figure S11.** Simulated predicted (blue) and actual (orange) test spectra from the drinking water data set from CNN regression model.

**Figure S12.** Difference between CNN regression predicted and actual test spectra from the drinking water data set.

**Figure S13.** Simulated predicted (blue) and actual (orange) test spectra from the sparse drinking water data set from CNN regression model.

**Figure S15.** Difference between CNN regression predicted and actual test spectra from the sparse drinking water data set.

**Figure S16.** Simulated predicted (blue) and actual (orange) test spectra from the river water data set from CNN regression model.

**Figure S17.** Difference between CNN regression predicted and actual test spectra from the river water data set.

**Figure S18.** Simulated predicted (blue) and actual (orange) test spectra from the drinking water sparse data set from CNN regression model.

**Figure S19.** Difference between CNN regression predicted and actual test spectra from the sparse river water data set.

**Target Concentration Prediction**

The concentration of each pollutant was predicted by dividing the first 5 label predictions by the last label, the predicted normalization factor. Then each normalized pollutant contribution was scaled by the maximum height of the unnormalized, blank and background-subtracted test spectrum. Then the target concentration was found by converting each scaled label based on the exponential calibration curve. All resultant negative values were set to 0.
